# Identification of Resectable N2 in NSCLC: A Single Center Experience and Review of the SEER Database

**DOI:** 10.3389/fonc.2021.647546

**Published:** 2021-04-26

**Authors:** Yan-qing Wang, Xu-dong Liu, Wen-liang Bai, Shan-qing Li

**Affiliations:** ^1^ Department of Thoracic Surgery, Peking Union Medical College Hospital, Chinese Academy of Medical Sciences & Peking Union Medical College, Beijing, China; ^2^ Medical Science Research Center, Peking Union Medical College Hospital, Chinese Academy of Medical Sciences & Peking Union Medical College, Beijing, China

**Keywords:** NSCLC, SEER, resectable, nomogram, prognosis

## Abstract

**Background:**

Non-small cell lung carcinoma (NSCLC) with ipsilateral and/or subcarinal mediastinal lymphatic spread (N2) is a heterogeneous disease. The role of surgical resection in patients with N2 NSCLC remains controversial and no survival-based definition of “resectable N2” exists. The purpose of this study is to evaluate the factors that potentially affect the survival of N2 NSCLC patients who receive surgical resection and to define “resectable N2” based on the survival benefits.

**Methods:**

Data from the open Surveillance, Epidemiology, and End Results (SEER) database from the National Cancer Institute in the United States were used to construct a nomogram. Patients who received surgery between 2010 and 2015 for N2 NSCLC were included. Independent prognostic factors for survival identified through Cox regression analysis were used to create the nomogram. The C-index, receiver operating characteristics (ROC) analyses, calibration curves, and risk stratification were used to evaluate the nomogram. The nomogram was also validated using data from 222 patients from Peking Union Medical College Hospital (PUMCH). Furthermore, lung cancer–related deaths were compared using competitive risk analysis.

**Results:**

In total, 4267 patients were included in the SEER cohort. Male gender, old age, high T stage and grade, adenosquamous and squamous cell carcinoma, lower lobe and overlapping lesions, extended lobe or bilobectomy and pneumonectomy, no chemotherapy, radiation before and after surgery, positive number of lymph nodes, and lymph node ratio (LNR) were identified as independent risk factors for higher mortality. The nomogram was created using these parameters. The C-index was 0.665 (95% confidence interval (CI), 0.651-0.679) and 0.722 (95% CI, 0.620-0.824) in the SEER and PUMCH cohorts, respectively. The calibration curves showed satisfactory consistency between the predicted and actual survival status in both the SEER and PUMCH cohorts. Competitive risk analysis confirmed that the variables in the nomogram, except radiation, are risk factors for prognosis.

**Conclusions:**

“Resectable N2” should be assessed by a multidisciplinary team. The novel nomogram developed in this study may help with clinical decision-making for this patient population.

## Background

N2 refers to lung cancer metastasis in ipsilateral mediastinal lymph nodes, which accounts for approximately 20%-30% of all non-small cell lung carcinoma (NSCLC) (1, 2). Although many treatment strategies (bimodality or trimodality) exist for N2 NSCLC, the effectiveness of these therapeutic strategies remains unsatisfactory and the 5-year survival rate ranges from 23%-36% (3, 4). Patients with N2 NSCLC are a notoriously heterogeneous population with variable clinical outcomes and choosing the correct treatment strategy with or without surgery remains a challenge for clinicians.

For bulky N2, defined as mediastinal lymph nodes that have a short-axis diameter greater than 2 cm with signs of invasion in surrounding tissues on chest computed tomography (CT), positive surgical margins are highly likely, and therefore, the consensus is to refrain from surgery (5–7). However, some N2 are resectable. Resectable N2 refers to discrete lymph nodes with a short-axis diameter less than 2.5-3 cm with no extranodal extension into adjacent tissue structures (6, 8, 9). And it needs thorough pathological nodal staging completed (10). However, even for this type of N2, there is still much controversy over whether surgery is appropriate. There is no survival-based definition of what constitutes a “resectable” N2 tumor. The decision to perform or forego surgery is often made by a surgical team by examining the tumor boundaries with contrast CT.

In clinical practice, experience has shown that patients with pathology-proven N2 (pN2) who undergo surgery can have drastically different outcomes, and the 5-year overall survival rate was varied from 35%-76% (11). This might be due to N2 disease heterogeneity. Therefore, we hypothesized that the decision to perform or forego surgery should not be based only on the morphological characteristics of the lymph nodes on CT and other clinical and pathological factors that can affect prognosis should also be taken into consideration. Additionally, “resectable N2” should be defined in terms of the survival benefits of resection.

This study was conducted to identify the prognostic factors that affect survival in patients with pN2 who undergo surgery and to define “resectable N2” for NSCLC patients. We used the open Surveillance, Epidemiology, and End Results (SEER) database from the National Cancer Institute in the United States to develop a nomogram aimed at assisting multidisciplinary teams in predicting individual prognosis and improving clinical decision making for patients with N2 NSCLC. The nomogram was validated with data from the Peking Union Medical College Hospital (PUMCH).

## Methods

### Training Cohort and Data

We retrieved data from the SEER database using the SEER*STAT 8.3.6 software. A custom data file was obtained from the SEER Program with permission number 15674-Nov2019. Inclusion criteria included the following: diagnosis in 2010-2015, patients underwent surgery and pN2.

**Figure 1:**
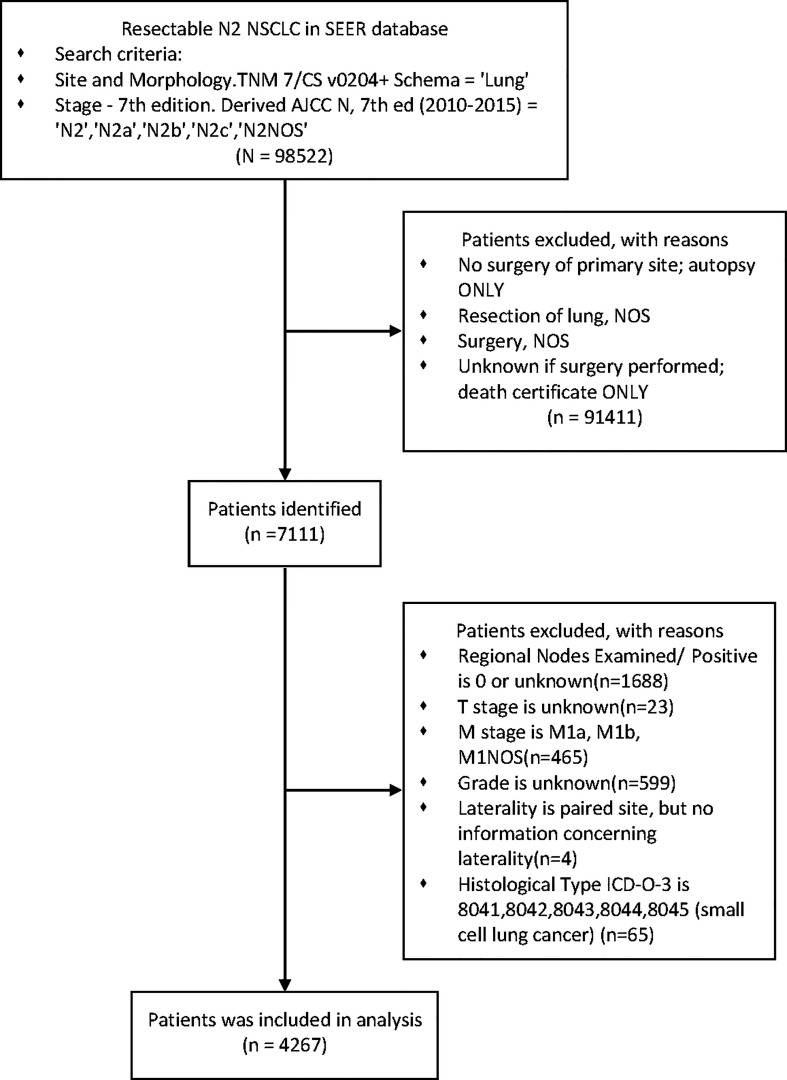
Flow diagram of patient selection in the SEER database.

Exclusion criteria were as follows: incomplete information recorded, any M1 and small cell lung cancer. (**Figure 1**)

Variables extracted from the SEER database included sex, age at diagnosis, race, marital status, primary tumor site, laterality, ICD-O-3 histology code and behavior, pathologic grade, American Joint Committee on Cancer T/M stages, pathological nodal staging, positive regional nodes, regional nodes examined, radiation sequence with surgery, chemotherapy recode, survival months, vital status recode, cause of death (COD) site recode, SEER specific death classification, and SEER other COD classification. The lymph node ratio (LNR) was obtained by dividing the number of positive regional nodes by the total number of lymph nodes examined (12). The primary outcome was overall survival (OS). The secondary outcome was NSCLC specific survival.

### External Validation Cohort and Data

To validate the developed nomogram in a responsible manner, an external validation cohort treated from March 2016 to July 2019 in the Department of Thoracic Surgery at PUMCH was used. The study was approved by the PUMCH Ethical Committee (No. B260). The cohort included 222 postoperative pN2 NSCLC patients. The inclusion and exclusion criteria were the same as those for the training cohort from the SEER database. The last follow-up for the cohort was in Aug 2020 and the primary outcome was also OS.

### Construction and Evaluation of Prognostic Model

We performed a univariate Cox proportional hazard regression analysis in a forward stepwise manner to identify possible independent prognostic factors. Factors found to be statistically significant in the univariate analysis were used in a multivariate analysis to estimate the hazard ratio (HR) and corresponding 95% confidence intervals (CIs) for every independent prognostic variable. The prognostic nomogram was created based on univariate Cox proportional hazard regression analysis using R packages (“survival, rms”) (13).

To evaluate the nomogram, C-statistics was used to evaluate overall discrimination of the nomogram. And the AUC was used to evaluate discrimination of the nomogram at the given time(6-month, 1-year, 3-year) (14). The C-index ranged from 0.5 (no discrimination) to 1.0 (perfect discrimination). Calibration accuracy was measured with a calibration curve to determine how close the predicted probabilities were to the actual survival outcomes. The calibration curves of the nomogram for 0.5, 1, and 3-year OS were plotted with the R package (“survival, survivalROC, rms”) (15, 16). for both the training and validation cohorts. All evaluation processes were performed by bootstrapping (1000 repetitions).

To further evaluate the prognostic model, the cohort was divided into two risk groups (low and high) according to the prognostic scores in the nomogram. Log-rank survival analysis was used to identify differences in survival between the low and high-risk groups. The discrimination ability of the nomogram was evaluated using the “survminer” package (17).

### Competitive Risk Analysis of Cancer-Related Deaths

In addition, according to the COD code, we classified the COD into two groups: NSCLC-related death and Other-related death. We also performed competing risk analysis with the Fine & Gray model using the”cmprsk” R package to reduce the possible impact of competing risk bias, which can be significant in multivariate Cox proportional hazard regression analysis (18).

Statistics and graphing were performed using Rstudio 1.2.5003. Continuous variables were tested using two-tailed t-tests, and categorical variables were tested using chi-square tests. Mann-Whitney test was applied for nonparametric data. P<0.20 was considered statistically significant in univariate analysis. Otherwise, P<0.05 was considered statistically significant.

## Results

### Clinicopathological Characteristics of Training and Validation Cohorts

After stepwise selection, the training cohort included 4267 cases from the SEER database and the external validation cohort included 222 cases from PUMCH. The demographics and clinicopathologic characteristics are summarized in **Table 1**.

**Table 1:** Demographics and Clinicopathologic Characteristics of Patients underwent surgery with pN2.

Demographic or Characteristic	SEER Cohort (n = 4267)		PUMCH Cohort Cohort (n = 222)		P value
	No. of Patients	%	No. of Patients	%	
**Age at diagnosis**					0.570
Median (IQR)	67(60-74)		61(54-68)		
Range	16-94		23-81		
**Sex**					
Female	2132	50.0	109	49.1	0.801
Male	2135	50.0	113	50.9	
**Race**					
Black	408	9.6	–		
White	3505	82.1	–		
other	354	8.3	–		
**Marital status**					
Married	2532	59.3	–		
Unmarried	1735	40.7	–		
**Histology**					**<0.001**
Adenocarcinoma	2801	65.6	178	80.2	
Squamous cell carcinoma	965	22.6	32	14.4	
Adenosquamous carcinoma	137	3.2	6	2.7	
Neuroendocrine tumors	191	4.5	4	1.8	
NSCLC NOS	86	2.1	0	0	
Others	87	2.0	2	0.9	
**Histologic grade**					**<0.001**
Well differentiated	289	6.8	12	6.8	
Moderately differentiated	1836	43.0	163	73.4	
Poorly/Undifferentiated	2142	50.2	44	19.8	
**Laterality**					0.417
Left	1906	44.7	93	41.9	
Right	2361	55.3	129	58.1	
**Primary Site**					**0.015**
Main bronchus	47	1.1	0	0	
Upper lobe	2398	56.2	105	47.3	
Middle lobe	221	5.2	16	7.2	
Lower lobe	1453	34.0	93	41.9	
Overlapping lesion of lung	85	2.0	3	1.3	
Lung&Bronchus, NOS	63	1.5	5	2.3	
**T stage**					**<0.001**
T1	1087	25.5	100	45.1	
T2	2053	48.1	108	48.6	
T3	811	19.0	8	3.6	
T4	316	7.4	6	2.7	
**Type of surgery**					**<0.001**
Resection of less than one lobe	458	10.7	5	2.3	
Resection of [at least one] lobe or bilobectomy	301	7.1	23	10.3	
Lobectomy with mediastinal lymph node dissection	2977	69.8	189	85.1	
Lobe or bilobectomy extended	148	3.5	0	0	
Pneumonectomy	383	8.9	5	2.3	
**chemotherapy**					0.005
No/Unknown	1065	24.8	37	16.7	
Yes	3202	75.2	185	83.3	
**Radiation**					**<0.001**
No	2328	54.6	177	79.7	
RS	2	0.0	0	0	
R+S	349	8.2	1	0.5	
S+R	1554	36.4	44	19.8	
R+S+R	34	0.8	0	0	
**Lymph nodes examined, number**					**<0.001**
Median (IQR)	11(6-17)		20(15-27)		
Range	1-90		2-50		
**Positive lymph nodes, number**					
Median (IQR)	2(1-5)		4(2-7)		**<0.001**
Range	1-61		1-34		
**LNR**					
Median (IQR)	0.250(0.125-0.5)		0.200(0.110-0.380)		**<0.001**
**NSCLC related death**	1785	41.8	33	14.9	
**Other related death**	418	9.8	3	1.4	**<0.001**
**Alive**	2064	48.4	186	83.7	
**Follow-up, months**					0.241
Median (95%CI)	42 (41-44)		31(29-33)		
Range	0-83		5-53		

RS, Intraoperative rad with other rad before/after surgery.

R+S, Radiation prior to surgery.

S+R, Radiation after surgery.

R+S+R, Radiation before and after surgery.

LNR, Lymph node ratio. In the p value column, the bolded values mean that they have significant difference.

There were no significant differences in the age at diagnosis, sex, and laterality distribution between the two cohorts. However, the histology type and grade distribution differed. Compared to the SEER cohort, the PUMCH cohort included a higher proportion of patients with adenocarcinoma (80.2% vs 65.6%), and a smaller proportion of poorly differentiated or undifferentiated tumors (19.8% vs 50.2%). The PUMCH cohort also included more patients with T1/2 stage disease. More lymph nodes were examined in the PUMCH cohort than in the SEER cohort and more metastatic lymph nodes were also found. The median LNRs were 0.20 in the PUMCH cohort and 0.25 in the SEER cohort. The patients in the PUMCH cohort also received different treatments compared to those in the SEER cohort. More patients underwent lobectomy with mediastinal lymph node dissection (LM) in the PUMCH cohort (85.1%) compared to the corresponding proportion in the SEER cohort (69.8%) and fewer underwent pneumonectomy (2.3% vs 8.9%). In the PUMCH cohort, a higher proportion of patients received chemotherapy (83.3% vs 75.2%) and a lower proportion received radiotherapy regardless of the sequence with surgery (20.3% vs 45.4%).

### Risk Factors for OS

There were 1785 events (NSCLC-related deaths) in the SEER cohort and the median follow-up period was 42 months (95%CI:41-44 months) (Reverse Kaplan Meier method). According to the results of univariate Cox regression analyses, age, sex, race, histology type, grade, tumor locations, T stage, surgery, chemotherapy, radiotherapy, number of positive lymph nodes, and LNR were all significantly associated with OS. Marriage, laterality, and number of examined lymph nodes did not significantly affect OS. All 12 significant factors were entered into a multivariate Cox regression analysis, which revealed the independent prognostic factors (**Table 2**). Male sex, old age, high T stage and grade, adenosquamous and squamous cell carcinoma, lower lobe and overlapping lesions, extended lobe or bilobectomy and pneumonectomy, no chemotherapy, radiation before and after surgery, number of positive lymph nodes, and LNR were found to be associated with a higher risk of death.

**Table 2:** Cox regression analysis of overall survival of patients underwent surgery with pN2 in SEER cohort.

Variables	Univariate HR (95% CI)	P Value	Multivariate HR (95% CI)	P value
**Age at diagnosis**	1.024(1.019-1.029)	**<0.001**	1.019(1.014-1.025)	**<0.001**
**Sex**				
Female	Ref			
Male	1.384(1.261-1.519)	**<0.001**	1.326(1.204-1.461)	**<0.001**
**Race**				
Black	Ref			
White	1.210(1.025-1.428)	**0.024**	1.122(0.949-1.327)	0.178
other	1.096(0.872-1.377)	0.432	1.049(0.833-1.322)	0.683
**Marital status**				
Married	Ref		–	
Unmarried	1.021(0.929-1.122)	0.673		
**Histology**			–	
Adenocarcinoma	Ref			
Squamous cell carcinoma	1.234(1.105-1.377)	**<0.001**	1.149(1.021-1.293)	**0.021**
Adenosquamous carcinoma	1.377(1.078-1.758)	**0.010**	1.363(1.063-1.745)	**0.014**
Neuroendocrine tumors	0.687(0.523-0.902)	**0.007**	0.696(0.526-0.920)	**0.011**
Nsclc NOS	1.037(0.744-1.445)	0.832	0.918(0.656-1.284)	0.616
Others	1.354(0.992-1.849)	**0.056**	1.190(0.867-1.631)	0.281
**Histologic grade**				
Well differentiated	Ref			
Moderately differentiated	1.237(1.000-1.529)	**0.049**	1.140(0.918-1.416)	0.236
Poorly/Undifferentiated differentiated	1.567(1.271-1.932)	**<0.001**	1.404(1.132-1.742)	**0.002**
**Laterality**				
Left	Ref		–	
Right	1.051(0.957-1.154)	0.299	–	
**Primary Site**				
341 Upper lobe	Ref			
340 Main bronchus	1.472(0.955-2.268)	**0.079**	1.167(0.737-1.848)	0.510
342 Middle lobe	0.908(0.725-1.136)	0.397	0.931(0.743-1.165)	0.528
343 Lower lobe	1.200(1.086-1.326)	**<0.001**	1.144(1.033-1.266)	**0.010**
348 Overlapping lesion of lung	1.640(1.217-2.212)	**0.001**	1.374(1.008-1.873)	**0.044**
349 Lung&Bronchus, NOS	1.200(0.833-1.747)	0.342	0.788(0.532-1.169)	0.237
**T stage**				
T1	Ref			
T2	1.269(1.125-1.433)	**<0.001**	1.213(1.072-1.373)	**0.002**
T3	1.796 (1.562-2.064)	**<0.001**	1.686(1.459-1.949)	**<0.001**
T4	1.542(1.275-1.866)	**<0.001**	1.489(1.217-1.823)	**<0.001**
**Type of surgery**				
Lobectomy with mediastinal lymph node dissection	Ref			
Resection of less than one lobe	1.301(1.124-1.507)	**<0.001**	1.153(0.984-1.351)	0.080
Resection of [at least one] lobe or bilobectomy	1.249(1.049-1.488)	**0.013**	1.172(0.982-1.398)	0.078
Lobe or bilobectomy extended	1.588(1.266-1.992)	**<0.001**	1.407(1.114-1.777)	**0.004**
Pneumonectomy	1.464(1.255-1.706)	**<0.001**	1.300(1.096-1.543)	**0.002**
**chemotherapy**				
No/Unknown	Ref			
Yes	0.669(0.604-0.742)	**<0.001**	0.705(0.628-0.791)	**<0.001**
**Radiation**				
No	Ref			
RS	8.141(1.142-58.021)	**0.036**	6.172(0.857-44.446)	0.071
R+S	0.727(0.604-0.874)	**<0.001**	0.932(0.766-1.134)	0.481
S+R	0.848(0.768-0.937)	**0.001**	0.899(0.807-1.001)	0.052
R+S+R	1.296(0.823-2.041)	**0.262**	1.865(1.175-2.960)	**0.008**
**Lymph nodes examined, number**	0.996(0.991-1.002)	0.173		
**Positive lymph nodes, number**	1.042(1.033-1.050)	**<0.001**	1.022(1.011-1.034)	**<0.001**
**LNR**	2.479(2.129-2.886)	**<0.001**	2.426(2.033-2.896)	**<0.001**

RS, Intraoperative rad with other rad before/after surgery.

R+S, Radiation prior to surgery.

S+R, Radiation after surgery.

R+S+R, Radiation before and after surgery.

LNR, lymph node metastatic ratio. In the p value column, the bolded values mean that they have significant difference.

### Prognostic Nomogram for OS

**Figure 2:**
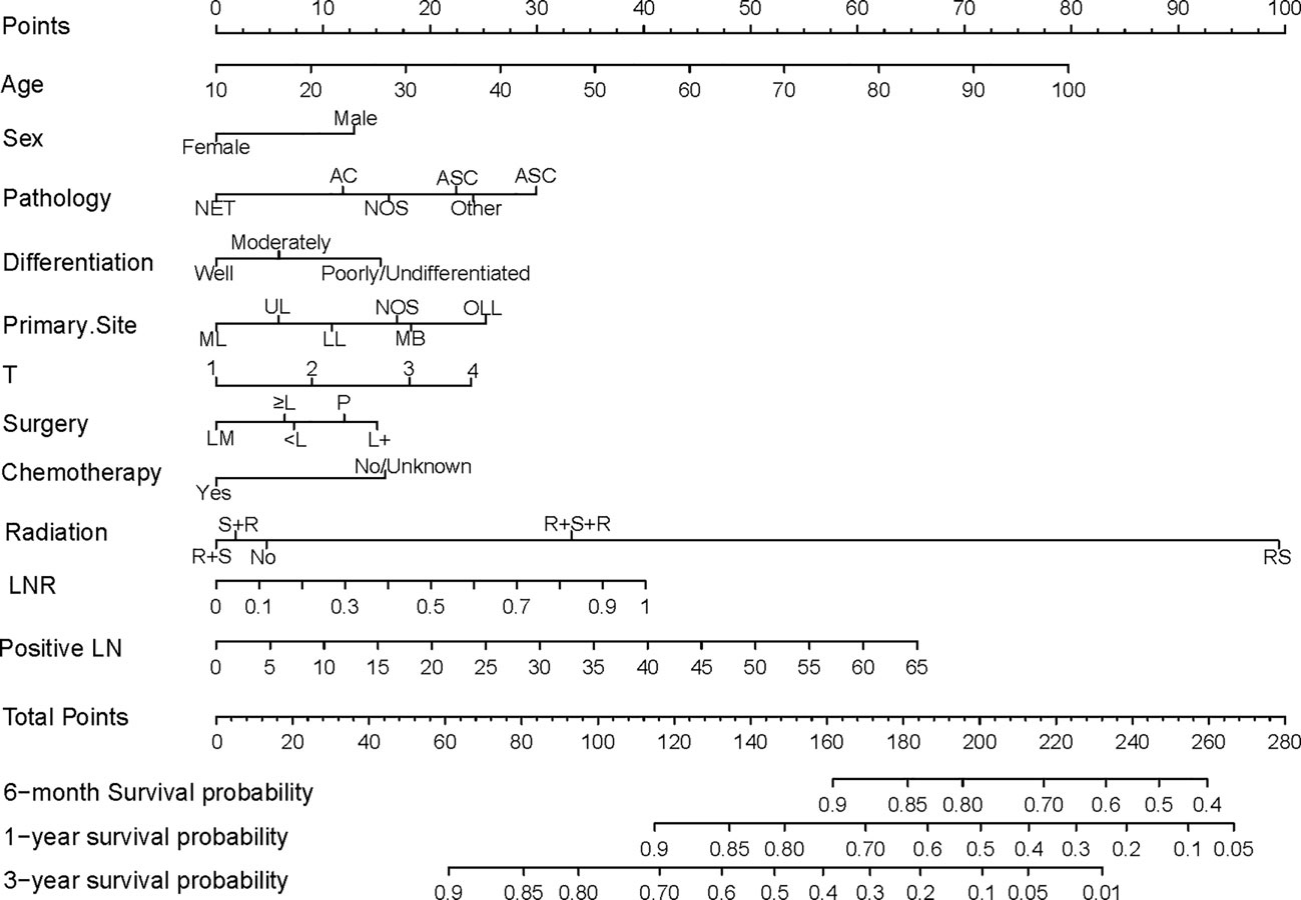
Nomogram predicting postoperative survival of pN2 NSCLC patients who underwent surgery. The value for each factor was located and a straight line was drawn upwards to the “points axis” of the nomogram to determine the number of points for the factor. The total number of points was calculated by summing the points for all factors. The value for each factor was found on the “total points axis” and a line was drawn straight downwards to the “survival axis” to estimate the probabilities of OS at 0.5, 1, and 3 years. AC, Adenocarcinoma; NET, Neuroendocrine tumor; SCC, Squamous cell carcinoma; ASC, Adenosquamous carcinoma; NOS, Not otherwise specified; ML, Middle lobe; UL, Upper lobe; LL, Lower lobe; MB, Main bronchus; OLL, Overlapping lung lesion; LM, Lobectomy with medial lymph node dissection; ≥L, Resection of [at least one] lobe or bilobectomy; <L, Excision or resection of less than one lobe; P, Pneumonectomy; L+, Lobe or bilobectomy extended; R+S, Radiation prior to surgery; RS, Intraoperative radiation with other radiation before/after surgery; R+S, Radiation prior to surgery; S+R, Radiation after surgery; R+S+R, Radiation before and after surgery; LN, lymph node number.

A nomogram was constructed using the independent prognostic factors to estimate 0.5-, 1-, and 3-year OS (**Figure 2**). The value for each factor was located and a straight line was drawn upwards to the “points axis” of the nomogram to determine the number of points for the factor. The total number of points was calculated by summing the points for all factors. The value for each factor was found on the “total points axis” and a line was drawn straight downwards to the “survival axis” to estimate the probabilities of OS at 0.5, 1, and 3 years (range 0.4-0.9, 0.05-0.9, and 0.01-0.9, respectively).

### Calibration and Validation of Nomogram

**Figure 3:**
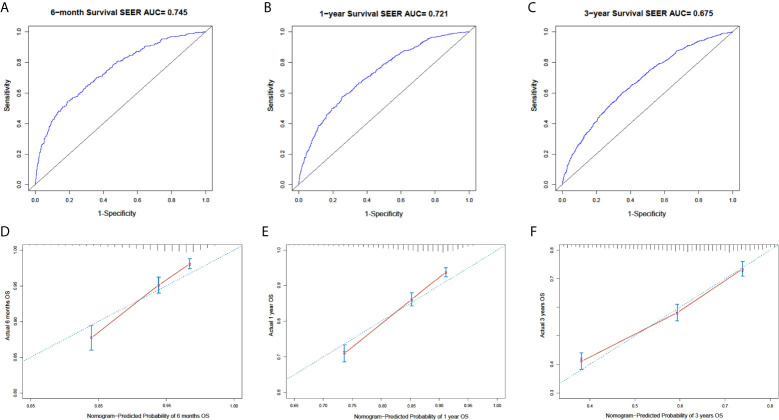
Nomogram with ROC analyses and calibration curves for training cohort. **(A–C)** Discrimination of 6-month (p < 0.01) **(A)**, 1-year (p < 0.01) **(B)**, and 3-year (p < 0.01) **(C)** OS in training cohort with ROC curves. **(D–F)** Calibration curves for 6-month **(D)**, 1-year, **(E)** and 3-year **(F)** OS from model. Y-axis and x-axis indicate actual survival probability and predicated survival probability, respectively. Blue dotted line indicates prediction accordance with actuality. Error bars show 95% CI.

**Figure 4:**
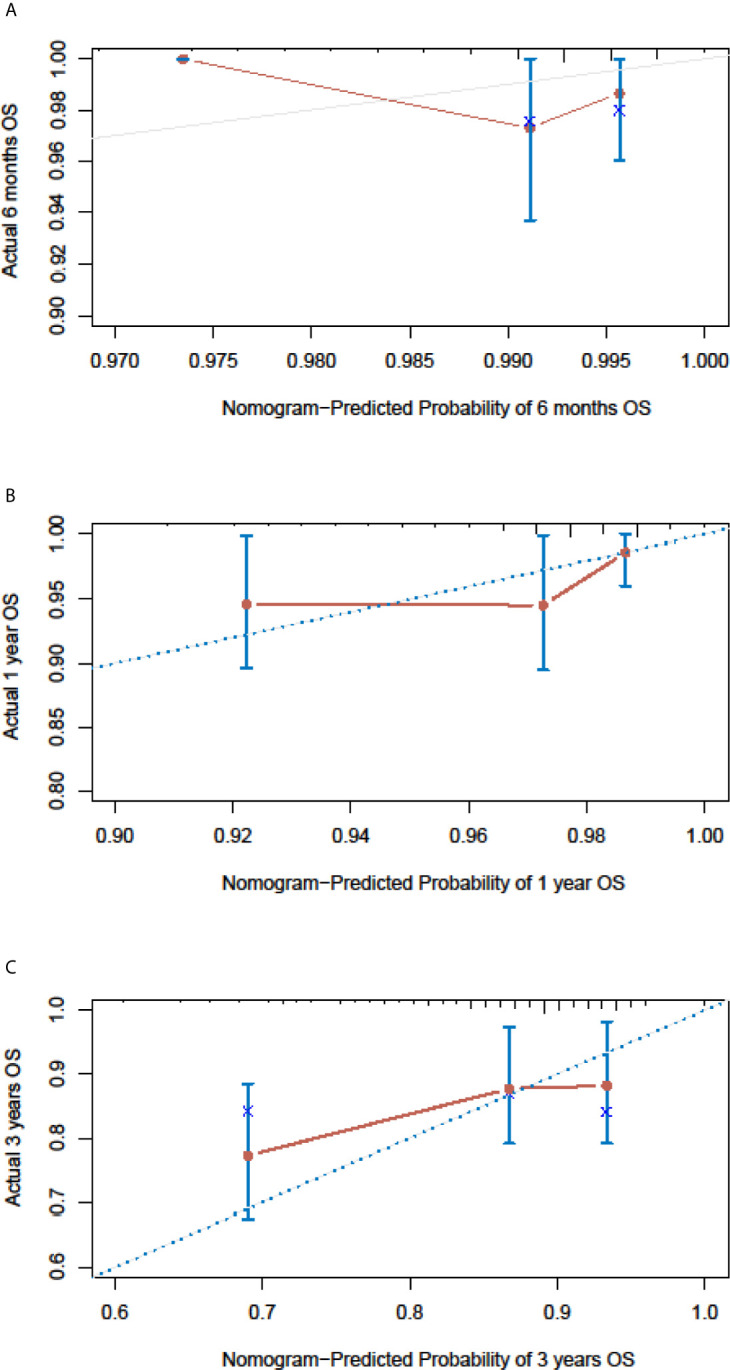
Nomogram calibration curves for the validation cohort. The calibration curves of the model for d 6-month **(A)**, 1-year **(B)** and 3-year **(C)** of overall survival in the validation cohort.

In the SEER cohort, the C-index was 0.665 (95%CI, 0.651-0.679) and the areas under the ROC curves (AUCs) were 0.674, 0.721, and 0.675, for 0.5, 1, and 3-year OS, respectively (**Figures 3A–C**). The calibration curves in **Figures 3D–F** show excellent consistency between the predicted and actual survival conditions in the SEER cohort, with the dots close to a 45° diagonal line (blue dotted line), thus, the nomograms were well calibrated. In the PUMCH cohort, the C-index was 0.722 (95% CI, 0.620-0.824) and the calibration curves also showed acceptable results (**Figure 4**).

**Figure 5:**
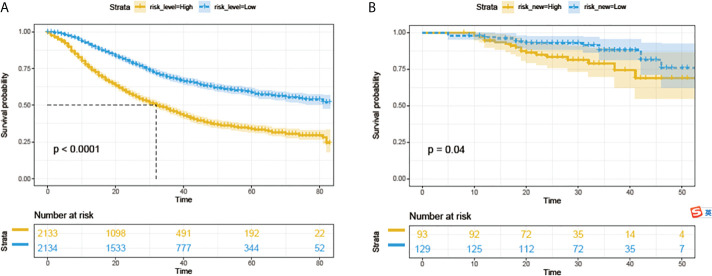
Survival analyses with log-rank risk stratification system for the SEER cohorts **(A)** and PUMCH cohort **(B)**. Yellow and blue lines show low-risk and high-risk groups, respectively.

All 4267 patients in the SEER cohort were divided into a high-risk group (n=2133) and a low-risk group (n=2134). High-risk patients had significantly worse OS than low-risk patients (**Figure 5A**). This result was further confirmed in the PUMCH cohort (**Figure 5B**).

### Competitive Risk Analysis NSCLC-Related Deaths

**Figure 6:**
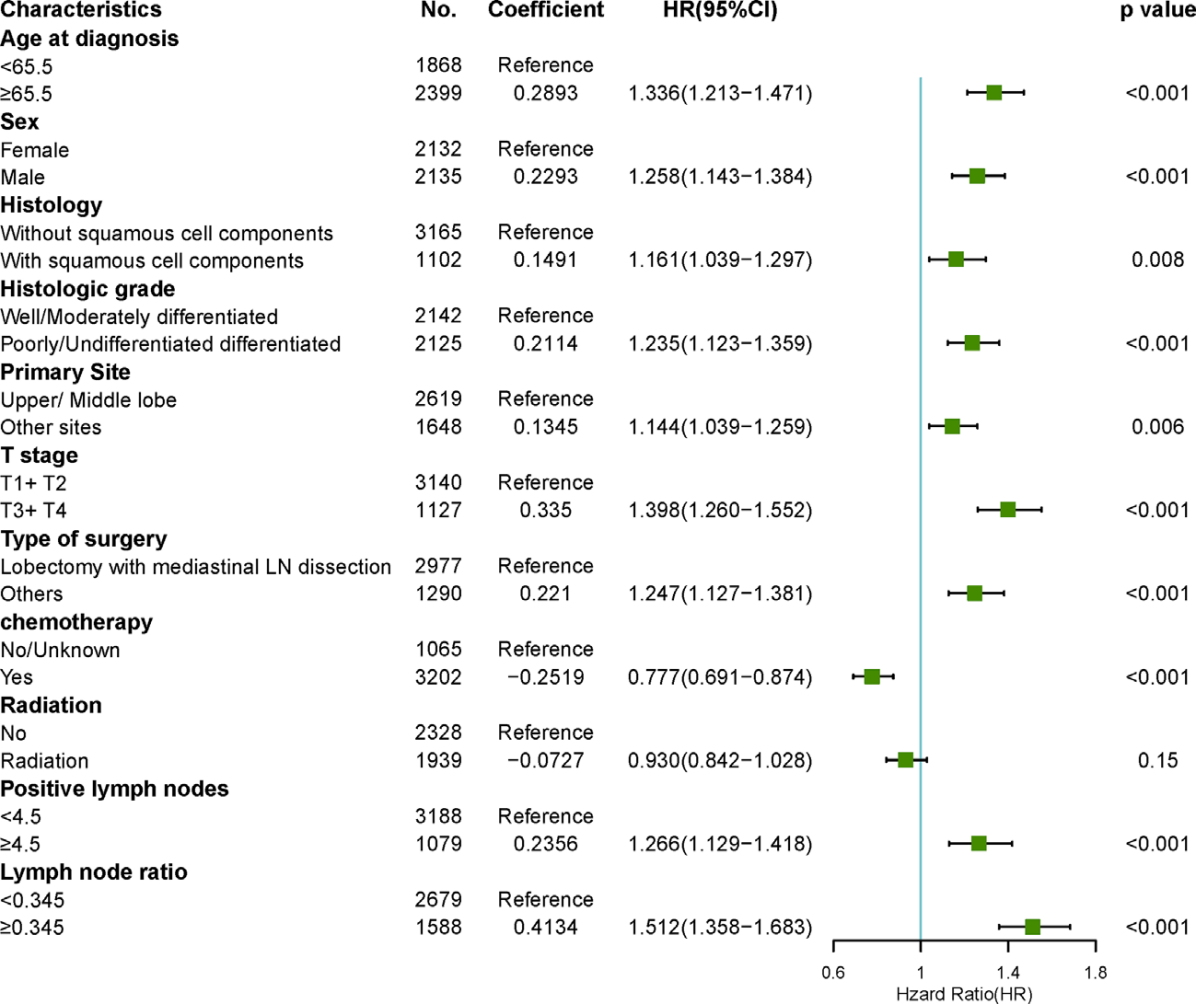
Forest plots visualizing the hazard ratios of clinicopathological characteristics for lung cancer-specific mortality in patients with pN2 who underwent surgery using a multivariate competing risk model.

To reduce the impact of competing risk bias, we performed competing risk analysis using the Fine & Gray model to further assess the risks of the independent prognostic variables. To simplify clinical decision-making, we compressed independent prognostic variables into dichotomous variables. The results confirmed that age > 65, male sex, histology with squamous cell components, poor or no differentiation, sites other than the upper and middle lobes, T3/4, surgeries other than LM (lobectomy with mediastinal lymph node dissection), no chemotherapy, positive lymph nodes > 5, and LNR > 0.345 were risk factors for poorer prognosis. However, radiotherapy may be a protective factor for better prognosis, with a borderline p-value (**Figure 6**).

## Discussion

In this study, we retrospectively analyzed population–based data to construct a nomogram for risk factors that affect the OS of patients with N2 NSCLC to increase our understanding of “resectable N2” and assist multidisciplinary teams in clinical decision-making. The nomogram showed good discrimination and calibration accuracy. It also increased our understanding of the heterogeneous outcomes for N2 patients who undergo surgery and the clinicopathologic characteristics that affect the OS.

N2 NSCLC is a very heterogeneous disease with different prognoses and is treated with different therapeutic strategies. Whether pN2 patients should be treated with or without surgery remains a controversial question (19). Two randomized trials evaluated the effect of surgery in this patient population, but neither showed an OS benefit from surgery (20, 21). One possible reason for the negative results of the studies could be that these trials did not sufficiently take into account the heterogeneity of N2 NSCLCs (22).

*Evison M* (10) have proposed definition of “potentially resectable stage III NSCLC”, the definition included: 1) pathologically confirmed NSCLC; 2)Thorough pathological nodal staging completed (surgical or endoscopic); 3) Thorough radiological staging including at least PET-CT and MRI brain with contrast; 4) Primary tumor resectable with high probability of clear pathological margins and complete resection; 5) Any nodal disease is discrete, easily measurable and defined, free from major mediastinal structures including the great vessels and trachea with no individual lymph node measuring >3 cm. However, this definition did not consider the patients heterogeneous clinical characteristics exist. And some clinical character may affect surgery outcome.

In this study, only pN2 patients underwent surgery were included to create the nomogram which could be a bias to assist multidisciplinary teams in clinical decision-making before surgery. Although pN2 is not the same as cN2, there is some correlation between them. At present, the preoperative diagnosis of N2 is almost consistent with the pathological N2 according the enhanced CT and PET-CT.A meta-analysis assessed agreement between clinical N stage and pathologic N stage, the accordance between cN2 and pN2 is 67%(104/155), for these patients, before surgery the nomogram may assist multidisciplinary teams in clinical decision-making. And the study also showed the clinically overstaged patients (cN2, pN0-1) accounts for 30%(47/155), which also could be benefit from the surgery. For the clinical understaged patients (cN2, pN3), account for only 3% (4/155) (23). Actually, they have no indication for surgery, and the definitive systematic treatment is preferential. However these patients not the N2 patients and they are the real N3. So as the Matthew Evison proposed, “potentially resectable stage III NSCLC” need the thorough pathological nodal staging (10).

N2 NSCLCs are advanced and systemic diseases. Accordingly, chemotherapy including targeted therapy is beneficial for patients with N2, both pre- or postoperatively (24, 25). And the number of chemotherapy cycles was an independent prognostic factor (11). Our results confirmed that chemotherapy is a significant protective factor for N2 patients who undergo surgery. If the surgical team considers the N2 resectable, the oncologist should assess whether the patient can tolerate full doses of neoadjuvant and/or adjuvant chemotherapy. Age, age-related comorbidities, which could influence chemotherapy tolerance, should be considered by the multidisciplinary teams.

To our surprise, we found that the primary site and surgery type could affect the prognosis. Similar results were also obtained in a published meta-analysis (26). In another study, the results showed no significant difference in OS between lesions located in the upper/middle and lower lobes (27). However, we noted that the majority of patients did not undergo surgery for lung tumor. So, for patients with N2 who underwent surgery, the primary site should be considerate. Patients who receive upper/middle lobectomy have larger remaining lung volume and thus have a higher probability of completing comprehensive treatment. Additionally, if the tumor is located in the main bronchus or when the lesions are overlapped, the remaining lung volume after pneumonectomy or bilobectomy is likely to be smaller. Therefore, the risk score is higher for patients who undergo pneumonectomy or bilobectomy. For N2 patients, the surgical team should estimate whether the remaining lung volume is enough for the patient to tolerate the systematic therapy that follows surgery. Additionally, central primary tumor location is associated with significantly worse outcomes (28). Consequently, if patients are more likely to receive pneumonectomy obtained R0 resection, mediastinal staging by mediastinoscopy or endobronchial ultrasound-guided aspiration is necessary, even for mediastinal lymph nodes ≤ 2 cm with clear boundaries.

LNR was reported as a predictor of OS and a useful complement to the N stage in patients with N2 disease status (29). And we confirmed the same results. However, it is very difficult to evaluate the number of lymph node metastases through non-invasive techniques such as PET-CT before the surgery. But we can fully evaluate the number of lymph node metastasis station preoperatively, which means that if multiple stations of metastasis occurred, LNR may be higher and the prognosis worse. Therefore, patients with multi-N2 station metastasis may not benefit from the surgery because of the higher LNR although surgeons believed that each lymph node could be removed.

It is undisputed that the T stage and tumor grade are significant independent predictive factors (30, 31). Besides these, we found that histology also affects prognosis. N2 patients without squamous cell components have better survival. Therefore, as many biopsies as possible should be performed if safety allows. The pathologist should also report the specific type of NSCLC and avoid the “NSCLC not otherwise specified (NOS)” designation as much as possible.

Lastly, to better identify “resectable N2” patients in the clinic, we created a nomogram to predict the individual survival rate. Variables in the nomogram were rated by a multidisciplinary team including surgeons, oncologists, pathologists, and radiologists. When applied to the external validation cohort, our model achieved considerable discrimination ability and calibration accuracy. Using the nomogram, we effectively stratified patients in the SEER or PUMCH cohort into two groups (high-risk and low-risk) with different OS. For high-risk patients, the effect of surgery is suboptimal, and these patients may not really have “resectable N2” although the size, shape, and borders of their lymph nodes may appear resectable to the surgeon. According to the nomogram, consideration all the risk factors that affect the OS, the appropriate selection of patients for surgery may avoid the operative risk of surgery in patients who may not benefit.

The study has some limitations. Firstly, we could not obtain all potentially relevant factors from the SEER database, such as preoperative mediastinal staging, the number of mediastinal stations of metastasis, patient comorbidities, length of progression-free survival, Eastern Cooperative Oncology Group (ECOG) performance scores, chemotherapy regimens, and molecular biomarkers. Further study is warranted to incorporate these variables into future research. Secondly, this was a retrospective study, not a randomized experiment, and therefore cannot establish direct cause-effect relationships. Thirdly, this study only included patients with pN2 who underwent surgery, and patients with no indications for surgery as well as those unwilling to undergo surgery were not included. Thirdly, the validation cohort size was not too much. Finally, although our model exhibits acceptable performance for separating patients into two groups, more research is necessary to compare treatment strategies with and without surgery in high-risk patient groups.

## Conclusion

N2 NSCLC is a systemic disease and its “resectability” should be assessed by a multidisciplinary team that not only takes into account the morphology of the lymph node on CT images, but also other clinical and pathological factors. This novel prognostic nomogram based on survival may be helpful for selecting patients with anatomically resectable N2 who will benefit the most from surgery.

## Data Availability Statement

The authors acknowledge that the data presented in this study must be deposited and made publicly available in an acceptable repository, prior to publication. Frontiers cannot accept an article that does not adhere to our open data policies.

## Ethics Statement

The studies involving human participants were reviewed and approved by the Peking Union Medical College Hospital (PUMCH) Ethical Committee (No. B260). The patients/participants provided their written informed consent to participate in this study.

## Author Contributions

Study concept and design: SL and YW. Data collection and assembly: WB and YW. Data analysis and interpretation: YW and XL. Manuscript preparation: YW. All authors contributed to the article and approved the submitted version.

## Funding

This work was supported by 2019 pumch science fund for junior faculty [grant numbers pumch201912058] and National Key Research and Development Program of China (Grant 2020YFB1313700).

## Conflict of Interest

The authors declare that the research was conducted in the absence of any commercial or financial relationships that could be construed as a potential conflict of interest.
